# Inactivation and Elimination of SARS-CoV-2 in Biosamples Using Simple Fixatives and Ultrafiltration

**DOI:** 10.3390/mps4010018

**Published:** 2021-02-27

**Authors:** Ranjeet Kumar, Afsal Kolloli, Selvakumar Subbian

**Affiliations:** Public Health Research Institute, New Jersey Medical School, Rutgers University, 225 Warren Street, Newark, NJ 07103, USA; rk879@njms.rutgers.edu (R.K.); ak1482@njms.rutgers.edu (A.K.)

**Keywords:** formalin, paraformaldehyde, ethanol, membrane filter, inactivation, plaque forming units, COVID-19, SARS-CoV-2, biocontainment

## Abstract

The Severe Acute Respiratory Syndrome-Coronavirus-2 (SARS-CoV-2) causes Coronavirus disease-2019 (COVID-19), which is an ongoing pandemic that has significantly affected the health, economy, and socio-economic status of individuals worldwide. Laboratory research using in vitro, ex vivo and in vivo models has been accelerated to understand the pathogenesis of SARS-CoV-2 infection. However, such experimental research involving SARS-CoV-2 is restricted to biocontainment/safety level-3 (BSL-3) settings, due to the high pathogenicity of this virus. Since many of the downstream analyses of SARS-CoV-2-infected biological samples need to be conducted in a non-BSL3 setting, it is important to ensure that the samples are fully decontaminated and safe for subsequent analysis. Here, we report the effectiveness of standard procedures used to fix cells and tissues for pathological analysis, including 2% or 4% paraformaldehyde, 50%–70% ethanol, 10% neutral buffered formalin and ultrafiltration using membranes with a molecular weight cut-off (MWCO) ranging from 3 to 30 kDa, for inactivating or eliminating SARS-CoV-2. We validated these methods in experimental laboratory samples, such as viral inoculum in cell culture media, SARS-CoV-2 infected host cells and animal tissue lysates. We found that 15 minutes’ treatment of viral inoculum (10^5^ plaque-forming units; PFU) or SARS-CoV-2 infected cells with paraformaldehyde or 70% ethanol resulted in complete inactivation of the virus. The treatment of infected hamster lung tissues with 10% neutral buffered formalin also fully inactivated the virus. However, only 3 kDa ultracentrifuge filter was effective in eliminating the virus to an undetectable limit in the filtrate. Our validated methods are useful for decontaminating biological samples to reduce infection risk and safe handling in BSL2 facilities.

## 1. Introduction

The ongoing coronavirus disease-2019 (COVID-19) pandemic, caused by a novel severe acute respiratory syndrome coronavirus-2 (SARS-CoV-2), has affected more than 93 million people and claimed about 2 million lives globally as of January 17, 2021 [1]. SARS-CoV-2 is an enveloped virus that is comprised of a single positive-stranded RNA genome and belongs to the genus Betacoronavirus [2]. Since there are no effective treatments available, research on various aspects of COVID-19 has gained considerable momentum to address the current emergency condition worldwide. Several experimental studies have been conducted on SARS-CoV-2 infection, pathogenesis, vaccine research and development of new treatment strategies [3,4]. Due to its potential zoonotic origin and ability to transmit through aerosol/droplet, to cause potentially life-threatening disease, SARS-CoV-2 is considered a strict BSL3 pathogen for research purposes [5,6].

Many basic works, such as the propagation of SARS-CoV-2 and the infection of cells and animals, should be performed at (BSL3) by specially trained personnel equipped with powered air-purifying respirators. To overcome this limitation, researchers use a pseudo virus that expresses SARS-CoV-2 surface protein for the development of in vitro assays [7]. Even though many laboratories have the facilities and expertise to work in a BSL3 lab, there is a lack of standardized protocols to work with the new virus. The inactivated samples have to be transported to lower biocontainment levels to carry out downstream studies, which includes the isolation of RNA from virus and virus-infected cells or tissue, characterization of viral genome sequences and to study pathogenesis and host response to infection. Moreover, inactivated virus and viral proteins are required for vaccine research and to characterize proteins for the development of antigen-based immunoassays. Few studies have reported the efficiency of various commonly used laboratory chemicals, such as solvents and detergents to decontaminate SARS-CoV-2, mostly in infected cells and culture supernatants [8,9,10,11]. Although these reports are broadly useful, the efficacy of common fixatives or membrane filters in sterilizing SARS-CoV-2 on infected animal tissues, has not been reported in these studies.

In the present study, we report the potential of paraformaldehyde, ethanol, 10% neutral buffered formalin and ultrafiltration, in inactivating SARS-CoV-2 in various types of infected biosamples. Each of these agents has been used to decontaminate biosamples containing pathogens handled in BSL3- and BSL4-settings, such as influenza and Ebola virus, in order to handle those samples safely in BSL2 facilities. We evaluated the decontaminating potential of various fixatives using viral inoculum, infected host cells and lung homogenates of SARS-CoV-2 infected hamsters. We used Vero E6 since SARS-CoV-2 propagates rapidly and efficiently in these cells as reported previously [12]. We used hamster lungs infected with SARS-CoV-2, since we wanted to validate the disinfection methods in a complex in vivo tissue, which is close to the natural niche, compared to cell line [13]. Furthermore, several pre-clinical vaccine and therapeutic studies of SARS-CoV-2 are conducted in animal models. Therefore, it is important to validate the disinfection procedures to render the in vivo biosamples safe to be handled in BSL2 facilities. The methods described in this study will allow researchers to select an appropriate inactivation method(s) for handling SARS-CoV-2-infected experimental laboratory samples in BSL-2 settings.

## 2. Experimental Design

### 2.1. Cell Culture and Propagation of SARS-CoV-2

The SARS-CoV-2-WA1/2020 strain was obtained from BEI resources (BEI Resources, Manassas, VA, USA). The virus was propagated in Vero E6 cells (African green monkey kidney epithelial cells; ATCC no. CCL-81) using Dulbecco’s Modification of Eagle’s medium (DMEM; Corning, Cat# 10-013-CV) containing 10% fetal bovine serum (FBS; Atlanta Biologicals, Flowery Branch, GA, USA, Cat # S11550). Briefly, 5 × 10^5^ Vero E6 cells were seeded into a 75 cm^2^ cell culture flask using DMEM containing 10% FBS. After 24 h of seeding (~85% confluency), the spent media was decanted and the monolayer was washed with sterile 1× PBS (pH 7.2; Corning, New York, NY, USA; Cat #21-031-CV) and, infected with 1 mL of SARS-CoV-2 (at multiplicity of infection 0.01–0.1) inoculum prepared in serum-free DMEM. The flasks were incubated at 37 °C for 1 h with gentle shaking every 15–20 min for virus adsorption. The infected cells were replenished with fresh DMEM containing 2% FBS and incubated at 37 °C with 5% CO_2_ for 48 h. The supernatant was centrifuged at 2000× *g* for 10 min at 4 °C, filtered through 0.45 μM filter and stored at −80 °C until use in a BSL-3 laboratory.

### 2.2. Infection of Syrian Golden Hamsters with SARS-CoV-2

Specific-pathogen-free, male Syrian golden hamsters (Envigo, Denver, PA, USA) that were 5 to 6 weeks old were infected with SARS-CoV-2 (10^6^ plaque-forming units (PFU) in 100 μL) by intranasal inoculation under sedation. At four days’ post-infection, hamsters were euthanized (n = 6) after deep sedation, and typical necropsy was carried out to collect organs for various assays. To determine the efficiency of fixation to inactivate the virus in the lung tissue, 100 mg of lung tissue was placed in 10% neutral buffered formalin (VWR, Radnor, PA, USA) and was kept for 24 h or 7 days. All procedures involving SARS-CoV-2 and hamsters were approved by the Institutional Animal Care and Use Committee (IACUC) and the Institutional Biosafety Committee (IBC) of the Rutgers University.

### 2.3. Removal of SARS-CoV-2 by Ultrafiltration

The Amicon^®^ Ultra Centrifugal filters (Millipore Sigma, St. Louis, MO, USA) with a molecular weight cut-of (MWCO) of 30 kDa, 10 kDa, 5 kDa and 3 kDa were used to determine their ability to filter out SARS-CoV-2 in various biosamples, such as viral inoculum in DMEM and supernatant from infected host cells. Briefly, 1 mL of biosample (from undiluted up to 6-logs dilutions) was added to the top compartment and centrifuged at 4500× *g* for 30 min at room temperature. In this method, >90% of the sample passes through the membrane into the collection vial. The centrifugation time was increased up to 1 h if less than 90% of the sample passed through the membrane. The filtrate in the bottom collection tube was carefully removed and virus infectivity titer was determined by plaque-forming unit (PFU) assay on serially-diluted filtrates and retentates.

### 2.4. Treatment of SARS-CoV-2 Infected Host Cells with Ethanol

The SARS-CoV-2 infected Vero E6 cells were treated with 50% and 70% ethanol for 15 min or 30 min at room temperature. After treatment, cells were washed three times with sterile 1× PBS and harvested. These cells were used to infect fresh Vero E6 cells and PFU assay was carried out in order to determine the effectiveness of ethanol treatment in inactivating the virus.

### 2.5. Treatment of SARS-CoV-2 Cell Culture Supernatant, Infected Host Cells and Lung Tissue Section with Formaldehyde

The supernatant from SARS-CoV-2 infected Vero E6 cells (6-well plate) were collected at 48 h’ post-infection. Both the virus containing supernatant and infected monolayer were treated with 2% or 4% paraformaldehyde or 10% neutral buffered formalin. The culture supernatants were dried on coverslip at room temperature in order to get rid of formaldehyde and PFU assay was carried out by seeding a monolayer of Vero E6 cells on top of the coverslips. To remove formaldehyde from treated VeroE6monolayers, cells were washed three times with sterile 1× PBS, harvested and used in the PFU assays. At specific time-points, lung tissues were washed five times in 10× volume of sterile 1× PBS to remove the residual formalin. Lung tissues were placed in a 2 mL microfuge tube with 1 mL DMEM medium and 0.5 mL beads and homogenized at maximum speed (6.5 m/s) for 2 min as 20 s pulses on a FastPrep unit (MP Biomedicals, Irvine, CA, USA). The tissue homogenate was centrifuged at 5000 rpm for 10 min at room temperature and clear supernatant was used for PFU assays. No cytotoxic effect was noted in the processed lung homogenates of uninfected hamsters, suggesting efficient removal of formalin.

### 2.6. SARS-CoV-2 PFU Assay

SARS-CoV-2 inoculum in DMEM media and virus-containing biosamples (i.e., cell culture supernatants, membrane-filtered cell lysates and tissue homogenates) were serially diluted using DMEM media with 2% FBS. The confluent monolayer of Vero E6 cells seeded in a 6-well culture plate (1 × 10^5^/well) (BD Biosciences, Franklin Lakes, NJ, USA) was washed three times with sterile 1× PBS and infected with 400 μL virus-containing samples (at various dilutions up to 10^−6^) and incubated at 37 °C/5% CO_2_ for an hour with gentle rocking every 15–20 min to prevent the monolayer from drying. Unabsorbed virus was removed by washing cells three times with sterile 1× PBS and was replenished with fresh minimal essential medium (MEM) containing agarose in each well. The MEM containing agarose was prepared by mixing an equal volume of 2× MEM (Thermo Scientific, Waltham, MA, USA; Cat No. 11935046) with 8% FBS and 1.6% low melting agarose (GE Biosciences, Niskayuna, NY, USA; Cat# 95057-712). The agar overlay was allowed to solidify at room temperature and the plates were incubated at 37 °C/5% CO2 for 48–72 h. A 4% paraformaldehyde (Thermo Scientific, Waltham, MA, USA, Cat # J19943-K2) solution was added to the top of the agar plugs in the plates and was incubated at room temperature for an hour. After removing formaldehyde, agar plugs were removed and the cells were washed three times with 1× PBS. The cells were stained with 0.2% crystal violet (Sigma-Aldrich, St. Louis, MO, USA; Cat #C0775) solution in 20% ethanol (Sigma-Aldrich, St. Louis, MO, USA; Cat #E7023-1L) at room temperature for 10 min and washed three times with sterile distilled water. The plaques in each well were enumerated and PFU per mL was calculated to the original inoculum. The PFU assays are capable of detecting even a single viral particle in the sample. We used multiple, serially-diluted samples for PFU assays and chose the dilutions that yield linear reduction in PFU to enumerate the virus.

All experiments were performed in triplicates (technical replicates) and in at least 2 sets of samples (biological replicates).

## 3. Results

### 3.1. Efficiency of Ultrafiltration Filters in Reducing Viral Titer in Culture Supernatants

**Figure 1:**
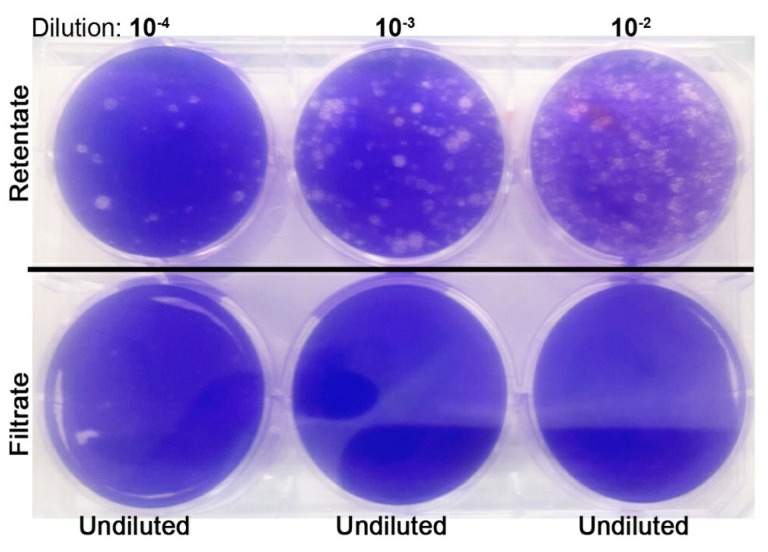
Representative image of plaque-forming units (PFU) assay performed on the retentate (top panel) and undiluted filtrate (bottom panel) of samples passed through 3 kDa molecular weight cut-off (MWCO) ultrafiltration unit. Note the viral plaques (white dots) only in the retentate.

Ultrafiltration methods using membrane-filter devices have been reported previously to concentrate viruses, including SARS-CoV-2 various sample types [8,9,14,15,16]. In those studies, Amicon^®^ Ultra-15 with a 30 kDa MWCO centrifugal filter device was used to concentrate the virus by centrifugation at 4500× *g* for 10 min at 4 °C. However, in our assays, we found a significant number of SARS-CoV-2 present in the filtrate after passing through filters with an MWCO of 30 kDa, 10 kDa and even 5 kDa (Table 1). In the PFU assays, we observed that the VeroE6 cell supernatant containing 5 × 10^5^ PFU/mL of SARS-CoV-2 was reduced to about 10 PFU/mL through filtration using a 30 kDa MWCO filter, while about 5 and 2 PFU/mL was noticed after filtration through 10 kDa and 5 kDa MWCO filters, respectively. However, ultra-filtration with a 3 kDa MWCO filter removed the viral particles to an undetectable level in our PFU assays (Figure 1).

### 3.2. Inactivation of SARS-CoV-2 by Formaldehyde Treatment

Formaldehyde is a commonly used as a fixative for host cells and tissues for downstream analysis such as histopathology and immunohistochemistry. We aimed to determine the effect of standard formaldehyde fixation conditions to inactivate SARS CoV-2 in the cell culture supernatant and infected cells. We found that treatment with 2% or 4% paraformaldehyde for 15 min or 10% neutral buffered formalin for 5 min was sufficient to inactivate the virus in both culture supernatants and infected Vero E6 cells (Table 2).

In SARS-CoV-2 infected hamster lung sections, we found that even one-day incubation with 10% buffered formalin have completely inactivated the virus as evidenced by lack of PFU in the treated tissue homogenates.

### 3.3. Inactivation of SARS-CoV-2 by Ethanol Treatment

To determine the effectiveness of ethanol in inactivating SARS-Co-V2, infected Vero E6 cells and the culture supernatants were treated with 50% and 70% ethanol for 15 or 30 min. We found that treatment with 70% ethanol for 15 min fully inactivated the virus; however, treatment with 50% ethanol up to 30 min was not sufficient to inactivate SARS-CoV-2 in biological samples (Table 2).

## 4. Discussion

Experimental biological specimens contaminated with SARS-CoV-2 must be handled in BSL3 settings, which poses significant challenges in analyzing the samples for various downstream applications, including flow cytometry, histology and enzyme-linked immunosorbent assay (ELISA). Therefore, it is very important and useful to validate commonly used chemical and mechanical inactivation methods to ensure safety while handling the samples in BSL-2 settings. In this study, we tested the efficacy of ultrafiltration and commonly used cell/tissue fixatives for the inactivation of SARS-CoV-2 in culture supernatants, infected host cells and animal tissue. The results indicate that ultrafiltration using 3 kDa MWCO is very efficient in filtering out SARS-CoV-2 from cell culture supernatants. Earlier studies have found ultrafilters to be effective in concentrating viruses from wastewater and other environmental samples [17,18]. However, for the first time, we have evaluated the efficiency of Amicon^®^ Ultra Centrifugal filters with different MWCO limits in eliminating SARS-CoV-2 from biological samples. The standard fixatives, including 2% and 4% paraformaldehyde, were found to be effective in inactivating infections of SARS-CoV-2 in 15 min at room temperature, whereas 10% buffered formalin was found to inactivate the virus in infected cells in 5 min. The efficiency of these fixatives and other reagents in inactivating SARS-CoV-2 was recently evaluated in culture supernatants and infected cells [8,9,10,11]. Our results on ethanol, paraformaldehyde and formalin to inactivate SARS-CoV-2 were consistent with, and supported by, these studies. However, we further evaluated the efficiency of fixative in inactivating SARS-CoV-2 in tissue samples. To summarize, our report adds information to the existing knowledge on procedures to decontaminate SARS-CoV-2 and contributes to developing standard guidelines for the safe handling of infected biosamples in BSL-2 settings.

## Figures and Tables

**Table 1:** Efficiency of ultrafiltration method in eliminating severe acute respiratory syndrome coronavirus-2 (SARS-CoV-2) from infected cell culture supernatants.

Amicon^®^ Ultra Centrifugal Filter MWCO	PFU/mL in Test Sample	PFU (min–max)/mL in Filtrate
30 kDa	5 × 10^5^	8–12
10 kDa	5 × 10^5^	3–7
5 kDa	5 × 10^5^	0–3
3 kDa	5 × 10^5^	Undetectable

MWCO-Molecular Weight Cut-Off; kDa-kilo Dalton; PFU-Plaque Forming Units. The experiments were done in triplicates using at least two independent biological samples.

**Table 2:** Efficiency of standard fixatives in eliminating SARS-CoV-2 from various sample types.

Reagent	Duration	Sample Type	PFU
2% Paraformaldehyde in 1× PBS	Not treated 15 min 30 min Not treated 15 min 30 min	Viral titer (positive control) Viral titer (treated) Viral titer (treated) VeroE6 cells infected with virus (positive control) VeroE6 cells infected with virus (Treated) VeroE6 cells infected with virus (Treated)	5 × 10^5^/mL 0 0 3 × 10^4^/mL 0 0
4% Paraformaldehyde in 1× PBS	Not treated 15 min 30 min	Viral titer (positive control) Viral titer (treated) VeroE6 cells infected with virus (Treated)	5 × 10^5^/mL 0 0
10% Neutral Buffered Formalin	Not treated 5 min 15 min 30 min Not treated 1 day 1 week	Viral titer (untreated-positive control) Viral titer (treated) VeroE6 cells infected with virus (Treated) VeroE6 cells infected with virus (Treated) Lung tissue (positive control) Treated tissue (1 Day) Treated tissue (1 Week)	5 × 10^5^/mL 0 0 0 3 × 10^7^/g 0 0
50% Ethanol	Not treated 15 min 30 min	VeroE6 cells infected with virus (positive control) VeroE6 cells infected with virus (Treated) VeroE6 cells infected with virus (Treated)	3 × 10^4^/mL 1-3/mL 0-3/mL
70% Ethanol	Not treated 15 min 30 min	VeroE6 cells infected with virus (positive control) VeroE6 cells infected with virus (Treated) VeroE6 cells infected with virus (Treated)	3 × 10^4^/mL 0 0

The experiments were done in triplicates using at least two independent biological samples.

## Data Availability

Not applicable.
